# Ketamine-Assisted and Culturally Attuned Trauma Informed Psychotherapy as Adjunct to Traditional Indigenous Healing: Effecting Cultural Collaboration in Canadian Mental Health Care

**DOI:** 10.3390/bs11090118

**Published:** 2021-08-31

**Authors:** Sherry-Anne Muscat, Geralyn Dorothy Wright, Kristy Bergeron, Kevin W. Morin, Courtenay Richards Crouch, Glenn Hartelius

**Affiliations:** 1 Youth Forensic Psychiatry, Alberta Hospital, Alberta Health Services, 17480 Fort Road, Edmonton, AB T5J 2J7, Canada; 2 Integral and Transpersonal Psychology, California Institute of Integral Studies, San Francisco, CA 94103, USA; ccrouch@ciis.edu (C.R.C.); ghartelius@ciis.edu (G.H.); 3 CreeAtion Community Care Society, Enoch Cree Nation, Chief Lapotac Blvd NW Suite 102, Enoch, AB T7X 3Y3, Canada; creeationcommunitycaresociety@gmail.com (G.D.W.); creeationconsulting@gmail.com (K.B.); 4 Department of Psychiatry, University of Alberta, 116 St & 85 Ave, Edmonton, AB T6G 2R3, Canada; kmorin@ualberta.ca; 5 Acute Adult Psychiatry, Alberta Hospital, Alberta Health Services, 17480 Fort Road, Edmonton, AB T5J 2J7, Canada

**Keywords:** trauma-informed care, culturally informed care, Indigenous health, ketamine, complex developmental trauma, colonialism, First Nations

## Abstract

Ketamine therapy with culturally attuned trauma-informed psychotherapy in a collaborative cross-cultural partnership may provide a critical step in the operationalization and optimization of treatment effectiveness in diverse populations and may provide a foundation for an improved quality of life for Indigenous people. Decolonizing Indigenous health and wellbeing is long overdue, requiring an equal partnership between government and Indigenous communities, built upon an aboriginal culture holistic foundation of balance of mind, body, social and spiritual realms, and within the context of historical and lived experiences of colonialism. Culturally attuned trauma-informed psychotherapy paired with ketamine—a fast-acting antidepressant that typically takes effect within 4 hours, even in cases of acute suicidality—may be uniquely qualified to integrate into an Indigenous based health system, since ketamine’s therapeutic effects engage multiple neuropsychological, physiological, biological, and behavioral systems damaged by intergenerational complex developmental trauma. Ketamine holds the potential to serve as a core treatment modality around which culturally engaged treatment approaches might be organized since its brief alteration of normal waking consciousness is already a familiar and intrinsic element of healing culture in many Indigenous societies. There is great need and desire in Indigenous communities for respectful and sacred partnership in fostering more effective mental health outcomes and improved quality of life.

## 1. Introduction

**Figure 1:**
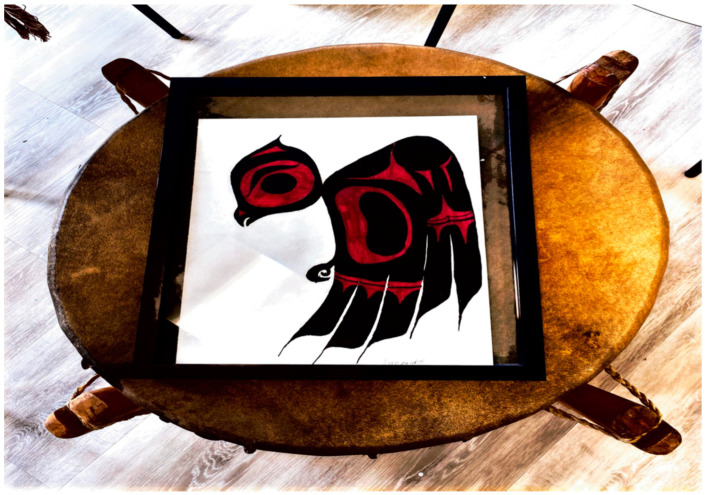
Sacred Medicine: Baby Owl on a Drum; The beginning story of self-reflection with the heartbeat of mother earth—Geralyn Dorothy Wright.

This is a call to action to address the burgeoning mental health crisis of Canadian Indigenous populations (Figure 1). This is a collaborative effort of frontline Indigenous and their allies’ clinical staff, modeling by example, and includes an Indigenous first-person perspective. In light of the discovery of unmarked mass graves of thousands of murdered and missing Indigenous children on the grounds of Canadian Indian Residential Schools across the country, implementing a clinical model grounded in traditional Indigenous healing has become more urgent. Indigenous people are grieving for their children that never came home, and for those who survived Indian Residential schools and other systematic forced assimilation atrocities, such as the 1960s scoop. We propose that ketamine-assisted culturally tuned, trauma-informed psychotherapy addresses some of the systemic mental health disparities of our Indigenous people, families, and communities suffering from the intergenerational complex trauma inflicted by Canadian colonialism.

The consequences of colonialism for Indigenous peoples in Canada are far-reaching, expansive, and cumulative—loss of culture and spirituality, loss of identity, broken families and communities, loss of oral history, loss of skills, traditions, and culture, loss of parenting abilities, attachment injuries, and emotional dysregulation. Mental illnesses such as chronic depression, anxiety, stress disorders, personality disorders, alcohol, and other substance disorders have triggered a mental health crisis as suicide rates for Indigenous youth soar to epidemic levels, exacerbated by poor coping skills and poor resilience. Other physical consequences include fetal alcohol spectrum disorder, diabetes, and autoimmune disorders. Systemic issues including institutional racism, physical abuse, sexual abuse, family violence, suicidality, neglect, poverty, and hopelessness, overrepresentation in the child welfare system, in the justice system, in gang life, and as victims of violent crimes. Without intervention, human suffering compounds. The offspring of Indian residential school survivors have less tolerance for life stressors than the generation before and have greater symptomology, suggesting a cumulative effect as numerous and sustained insults over generations combines with proximal stressors to undermine the collective wellbeing [1].

Countless official Canadian government inquiries into the challenge of addressing Indigenous health and wellbeing have resulted in thousands of sometimes highly effective pilot projects across the country, and yet, none have led to secure programing commitments [2,3]. Such undertakings deserve consultation with Indigenous representatives at a minimum and ideally full and equal partnership between government and Indigenous communities. Moreover, issues of Indigenous health need to be viewed within the historical and lived experience framework of colonialism [4]. There is a consensus amongst Indigenous scholars that wellness in native communities can only be achieved by decolonizing healthcare and perceiving wellbeing through a holistic aboriginal lens that enshrines balance between the mind, body, social, and spiritual realms. One approach to this goal may be through culturally attuned psychotherapy to address the underlying emotional wounds of Western colonialism, integrated with an Indigenous restorative health system of medicine wheel teachings, healing circles, ceremony, music, Indigenous traditional medicines, and shamanic healing.

## 2. Thesis Statements

Ketamine therapy may be a uniquely valuable adjunct to culturally integrative treatment approaches due to ketamine’s multi-layered biochemical efficacy, its promising potential when combined with trauma and attachment informed psychotherapy and its ability to induce mystical or peak experiences. 

Because of its therapeutic effects on neuropsychological, physiological, biological, and behavioral systems damaged by intergenerational complex developmental trauma, ketamine may even serve as a core treatment modality around which culturally engaged approaches might be organized.

The addition of cultural attunement may be the critical next step in the operationalization and optimization of treatment effectiveness across diverse populations and may provide a viable collaborative clinical model as a foundation for Indigenous people to benefit from the psychological growth and substantial improvement in quality of life that appropriate mental health care services can provide.

### 2.1. Ketamine Effects

Ketamine is a fast-acting antidepressant that typically takes effect within 4 hours, even for symptoms of acute suicidality, and is known to selectively induce neurogenesis, neuroplasticity, and improved brain connectivity in adults [5,6]. Ketamine also repairs the brain damage caused by chronic unpredictable stress depressed patients [7].

Ketamine alters normal waking consciousness, creating a brief (45 min) highly entropic brain state that may act as a restart button by disrupting the overly rigid negative self-beliefs, thought patterns, and worldviews associated with chronic depression [8]). At the same time, ketamine is a type of psychedelic, which is capable of inducing a profound and meaningful peak or mystical experience, described as a felt sense of happiness, wonder, mind expansion, and connectivity [9]; a feeling often lost to those suffering from chronic depression and other mental illnesses commonly associated with the generational trauma inflicted by colonialism.

Ketamine is a well-studied treatment for treatment-resistant depression, bipolar disorder, post-traumatic stress disorder, and alcohol and cocaine addictions. Ketamine appears to prolong abstinence from alcohol and heroin, as well as reduce craving for cocaine in non-treatment-seeking cocaine users [10]. Ketamine research is also expanding rapidly within the diverse fields of traumatic brain injury [11,12,13,14]. Degenerative brain disorders such as Parkinson’s and Alzheimer’s [15,16]. Neurodevelopmental disorders, including autism [17,18] and schizophrenia [5,6,19].

### 2.2. Altered States of Consciousness, Social Connectedness, and Indigenous Healing

Using altered states of consciousness for healing is already a familiar and intrinsic element of culture in many Indigenous societies. These states are typically achieved through the ingestion of Indigenous traditional medicines, fasting, sleep deprivation, and/or meditation and prayer. Although there is exploratory clinical use of psychedelics as an adjunct to psychotherapy in Western psychology examining the value of transformative experiences for mental disorders [20], this is not the norm in mental health systems. Perhaps because a holistic sacred approach to healing is less compatible with Westernized research methodologies that strive to measure the efficacy of individual factors and treatment applications that often center on one or two elements of intervention [3].

In many Indigenous cultures, altered states of consciousness enlighten relationships, foretell life pathways, and illuminate human interconnectivity of mind, body, spirit, community, earth, and the cosmos during individual knowledge-seeking—such as vision quest—and through community sacred ceremonies, such as sweat lodge and sun dance, both practiced by Cree and other First Nations people of Canada. Medicine people and elders serve as guides and interpreters for individuals and communities. They take this responsibility seriously and with great respect and humility [21]. There is evidence for the therapeutic efficacy of psychedelics in group settings, not only within Indigenous healing but also in Western psychiatric clinical practice, with a variety of mental diagnoses, including depression, anxiety, personality disorders, and alcoholism [22].

Within a culturally integrative approach, it might be preferable to incorporate Indigenous sacred traditional medicines with psychedelic effects rather than substituting a synthetic drug such as ketamine. While there is some preliminary research with peyote and psilocybin, most psychedelic substances, whether manufactured or in natural plant form, are highly regulated scientifically and medically in Canada and unlikely to change status anytime soon. Given these barriers to the incorporation of organic psychedelics, ketamine, as a legal substance with a long history of safety and efficacy with both mental and physical illnesses, appears to offer a pragmatic substitute that can function in what is often an essential role in traditional Indigenous healing, as well as function as an adjunct to culturally attuned psychotherapy.

Another of ketamine’s potential mechanisms of action is its effect of creating a sense of social connectedness [23]. This may help to explain its efficacy across a diverse range of mental health disorders, including a number of known challenges in Indigenous communities such as depression, anxiety, alcohol and drug addiction, and post-traumatic stress disorder [1]. Carhart-Harris et al. [24] hypothesized that social connectivity to others, nature, and the cosmos, and most importantly to oneself, and connectivity to past beliefs and past pleasures while promoting a sense of psychological wellbeing, peacefulness, and acceptance of one’s troubled past, is an underlying mechanism of psychological wellbeing (p. 547). This may be one way that Indigenous rituals and ceremonies create healing by engaging the whole person situated in a community and the world. Embracing all of the senses through music, smell, touch, sensation, ritual actions, and emotion, with elements integrated into a whole designed to facilitate a healing experience [25]. Ketamine’s impact on the sense of connectedness, when combined with Indigenous elements representing cultural belonging, may accelerate and intensify the healing process.

### 2.3. Insitu Evidence of Ketamine Assisted Trauma Informed Psychotherapy Efficacy

In a landmark retrospective study, Dore et al. [20] published affirmative outcome data from their successful community-based application of ketamine-assisted therapy. Participants were two hundred and thirty-five adult patients (mean age 42.7; 115 F, 120 M) from three distinct private general psychiatric practices located in Northern California. Notably, this was ketamine-assisted psychotherapy clinical data in situ, with real-world outcome data of the efficacy of ketamine-assisted psychotherapy on individuals diagnosed with a wide range of mental disorders common to a general psychiatry practice. This protocol may have the potential for adaptive generalization for Indigenous communities.

Dore et al. [20] reported significant decreases in anxiety and depression symptoms with a range of diagnoses. Subset analysis demonstrated that patients with (complex PTSD) or developmental trauma experienced the greatest improvement in depression and anxiety scores, whereas patients with the most severe symptom burden, including higher suicidality at intake, more frequent hospitalizations in the previous year, and higher aversive childhood events scales, had the most significant improvements across all measured domains. Furthermore, the authors concluded that psychedelic or dissociative effects were an integral part of the treatment and beneficial to patients when supported and integrated into a therapeutic context. Their results have positive implications for a successful integrative Indigenous-based, culturally focused health care system [20].

Of relevance to Indigenous healing, this protocol presupposes the importance of the relationship between treatment resistance, historical complex trauma, and the interpersonal neurobiology of the therapeutic alliance. According to the authors, breaking trust and lack of trust acquisition is the fundamental element of trauma persistence and treatment resistance. Therefore, tailoring the therapeutic alliance towards relationship bonding and trust building in a supportive, reliable, and compassionate environment that recognizes the patient’s vulnerable state is considered best practice in healing emotional wounds. Additionally, the therapist must be well versed in maintaining awareness of the neurobiological synchrony and attunement of the therapy relationship while also maintaining healthy boundaries that encourage psychological growth towards autonomy and self-reliance. This ketamine therapeutic model, as an adjunct to traditional healing, may be beneficial to the overall healing of Indigenous individuals, families, and communities [20].

Dore et al. [20] postulated that ketamine’s psychedelic effect, in conjunction with psychotherapy, increased ketamine’s efficacy by enhancing therapeutic communication and interaction. Furthermore, they observed that ketamine’s psychoactive properties shifted along a dose-related continuum; the ketamine-assisted psychotherapy protocol used this property to enrich the therapeutic process by escalating ketamine dosages at pivotal therapeutic moments, thereby accelerating the journey to psychological growth and wellbeing. Within this context, the authors developed a therapeutic modality based on what they termed trance and transformation. The trance state appeared to promote a time-out from the ordinary rigid, negative, and distorted cognitive processes associated with the depressive mind state, offering the individual relief from negativity and replaced it with a felt sense of openness and mind expansion. This enhanced the individual’s ability to engage in meaningful therapy, improve affect regulation, increase the ability to self soothe through meditation, process trauma, and promote depression recovery. The transformative state is a full out-of-body experience, which offers a similar but more intense therapeutic approach associated with the felt experience of connectivity and oneness whilst gaining a new perspective of oneself, and may be most effective when positioned as an escalating secondary phase of treatment.

This proposed collaborative paradigm may also serve to address another painful relic of colonialism, and that is the Western appropriation of Indigenous ways of being. New Age spirituality tends to appropriate and syncretize sacred rituals and ceremonies from various indigenous peoples without relational engagement or consent, separated from their Indigenous intentions and context of ceremony. Ultimately, this appropriation is a contributing factor to the erasure of the affected Indigenous cultures and only serves to add to their intergenerational trauma [25]. This collaborative model, together with a legal psychedelic such as ketamine as part of a comprehensive holistic Indigenous healthcare initiative, may be one opportunity, as an act of restoration, to benefit and empower Indigenous populations.

## 3. Collaborative Clinical Model

Traditional Indigenous healing is foundational in this collaborative model—provided by experienced practitioners of Indigenous ceremonies and other sacred medicines, teachings, and traditions with a commitment to healing and community, with an addition of the most advanced of Western medicine, technology, and practitioners, knowledgeable, progressive, and experienced. Staff should be well trained in psychedelic therapies, individual, family, and group psychotherapy, trauma-informed modalities, such as regulation and polyvagal theory, interpersonal neurobiology, and attachment injury, somatic methods, eye movement desensitization and reprocessing, trauma and dissociation, and ego-state and parts work. All clinic staff, (other than Indigenous) should be more than culturally competent: they should be allies. Furthermore, all medical staff, including the Indigenous people, should be fully educated in the systemic effects of colonialization on Indigenous people, as a society and as individuals, since what may look like cultural artifacts may well be reactive responding to the abuse suffered in a residential school [26].

This proposed collaborative clinical model should be founded on the Indigenous concept of healing ceremonies, sometimes referred to as medicine wheel or four directions teachings by Cree and other First Nations people in Canada. It must be a partnership with equal influence on all treatment decisions and serving the best interests of the people. Medicine wheel teachings stress that the pathway to health and wellbeing is through the mindful sacred attention to and attainment of balance and harmony amongst all aspects of our beings: mind, body, emotion, and spirit, with a sense of sacred oneness and unity of inner and outer worlds. An acknowledgment of lightness and darkness, a sense of one’s place and purpose—with nature, each other, spiritually, and with reverent connectedness with generation past and future. This is the ancient teachings, the Indigenous ways of being, or Walking the Red Road—a life path of strength, humility, and gratitude [27].

Medicine wheel teachings as a path to healing is not a new concept and has been successfully integrated into clinical practice in the past [27]. The main issue for Indigenous scholars is implementing an Indigenous developmental and ecological worldview framework while preventing the extraction of Indigenous knowledge by outsiders for the appropriation and control of Indigenous people. Indigenous scholars, researchers, and healers have a credible fear of being pushed aside and losing their voice once any project is implemented. The Canadian government has a history of doing just that and a poor track record regarding the protection of Indigenous people, communities, and ecosystems [27]. There is a justified lack of trust that can only be addressed in a truly collaborative healthcare model over time, wherein the Indigenous voice is not only heard but respected and has equal weight and value in policy, procedure, implementation, and treatment decisions. In fact, most treaties between Indigenous people and the Canadian government contain medicine chest clauses and guarantee treaty rights to health care for Indigenous people.

This model does not preclude Western medicine, but rather should embrace every advantage of incorporating all the cutting-edge knowledge and technology Western medicine has to offer, filtered through the medicine wheel or other Indigenous conceptual frameworks. Indigenous people have the right to access all innovative Western medicine and technology. A prime example is repetitive transcranial magnetic stimulation (rTMS), a relatively new treatment modality that is non-invasive and has good evidence of effectiveness with treatment-resistant depression [28]. With staff working together, anything is possible. This model is fluid and adaptive to specific community needs.

The advantage of this model of care will be in the mutual respect and partnership in a non-hierarchical collaboration, providing much-needed healing services. The clinic/healing center practice should mirror this approach. The clinic should provide individual, family, group, and community healing, integrating ketamine and trauma-informed psychotherapy protocols organically, within the presence of both Western and Indigenous practitioners, and as an adjunct to a combination of individual and group modalities that embrace traditional healing ceremonies. The clinic/healing center itself must be a safe, calm, and compassionate space for healing.

This clinic/healing center model is fluid and adaptive and can grow as community needs evolve, and trust and respect from the people and community are earned. It is possible for the clinic to evolve towards a community gathering place to share knowledge and skills and to help each other. This model offers the opportunity for community members to contribute, to teach lost skills, to learn, to connect socially, and to offer or receive needed services, such as child or elder care, or even home or vehicle repair. The possibilities are endless if services are provided within a collaborative, supportive, and safe environment—Indigenous history and culture, parenting groups, psychoeducation, addiction support groups, even traditional teachings for youth, such as the Pow Wow dance and drum or flute music. This could be an opportunity for the old to teach the young, to pass on knowledge, skills, and art. This model may enhance learning opportunities. For example, traditional food and modern nutrition, Indigenous traditional medicines and the science behind it, or meditation, yoga, and drumming. All these activities feed aspects of self that need attention to achieve balance and contribute to health and wellbeing through nurturing and care and by providing opportunities for life balance, connectivity, purpose, self-esteem and pride in culture, empowerment, and hope.

## 4. Discussion

Although further research of ketamine’s active properties is needed, in the quest for knowledge of the brain, the drug, and the dynamic interaction, it is apparent that ketamine’s maximum effectiveness is achieved when multiple mechanisms of actions are engaged. Ketamine induction appears to accelerate and mediate the psychotherapeutic process, and the psychotherapeutic process enhances ketamine efficacy and may increase the strength and duration of ketamine induction alone. Thus, it would be prudent and advantageous to view ketamine research, with its diversity of application, from an integral and transpersonal perspective focused on the known effects of ketamine when administered in conjunction with culturally attuned trauma-informed psychotherapeutic integration and interventions. Cultural attunement and an equal, respectful partnership collaborative model may in fact be the key to extrapolating and enhancing treatment effectiveness across any non-dominant marginalized communities.

Although this is a novel approach, this protocol is relatively safe and effective for clinical use, as all the individual components are known to be effective with substantial supporting evidence. Furthermore, there is an urgent need for the immediate implementation of a viable collaborative clinical model that encompasses both Indigenous and Western medicine in Canada. There may even be existing clinics that are able to adapt and implement this model immediately. Gathering scientific information and utilizing psychometric tools pre- and post-treatment would be most appropriate not only to collect efficacy data but also to modulate for targeted individualized care and adapt for different communities’ needs. The most appropriate and beneficial research and data collection strategy for this model might be an Indigenous participatory action construct in which the research process is as important as the outcome, and collaboration between researchers and participants is intrinsic to the model [29]. The ultimate objective of this proposal is to build a truly collaborative integrative pan-cultural approach that is transcultural, transpersonal, and rooted in the best scientific evidence available.

True to the framework of this collaborative Indigenous healthcare model, this proposal can only be authentic and meaningful if the discourse is situated within the context of this sacred partnership. This proposal was crafted in response to needs expressed directly by Indigenous people, as shared in the following section by our coauthors Geralyn Dorothy Wright and Kristy Bergeron, strong Indigenous women with lifelong dedication for Indigenous advocacy in Canada. 

## 5. Own Voices: Personal Narratives

The Truth Be Told, My Entire Being Longs for the Old Ways of My People.

## 6. My Story: Geralyn Dorothy Wright

**Figure 2:**
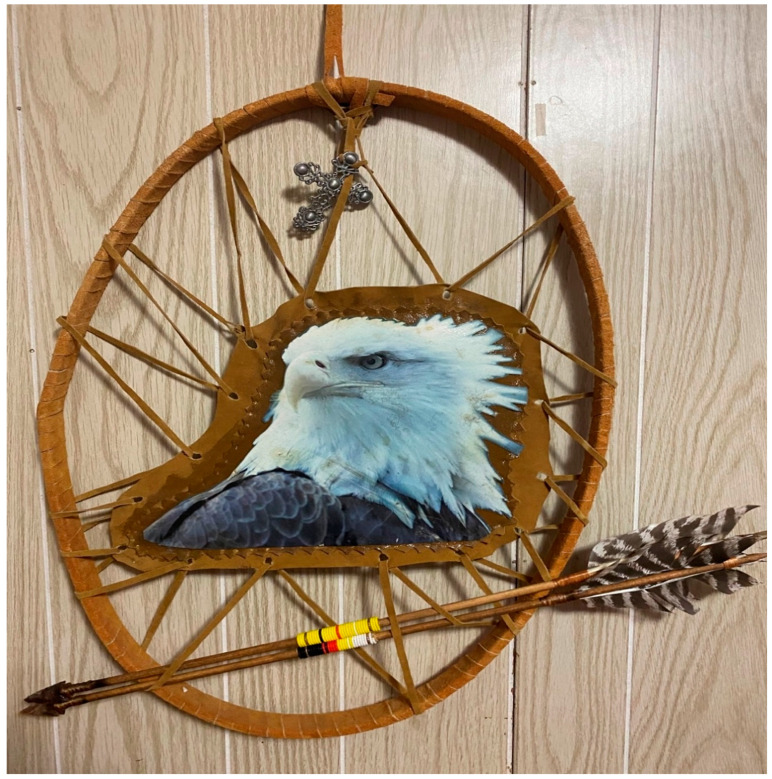
Sacred Medicine; Eagle on a Shield; Strength and protection, passed down through my mother—Geralyn Dorothy Wright.

“We are not Indians…we are not a problem”. We are the people of the land! (Figure 2).

To witness the generations speaking our Indigenous languages, to dance our traditional dances with the traditional understandings, to practice traditional ceremonies and walk this road with pride of who we are as Indigenous people! Finding our way through the shadows of Residential school and cultural genocide is the struggle.

My family is from Northern Alberta; my mother’s parents were from two neighboring Cree Communities in Treaty 8 land. Her parents, grandparents and their parents were all Cree from Northern Alberta, the Slave Lake region. They were hunters and gatherers, living off the land in a communal habitat where everyone was equal.

I recall the stories of the old people, of how it once was, many of my people have forgotten who they are and where we are from because of the experiences that have led up to this modern-day existence.

My Kokum would tell me stories about when she first saw a white-skinned person; she was maybe 10 years of age. She did not speak any English at that time, but the experiences that were felt were very scary and very confusing. These people brought to her home a new way of living, and they were very convincing with power that these ways were better.

Soon to be among the first to be taken to Residential school, my Kokum was taken away from her family, her community to be beaten and assaulted for being Cree. “Indians”!

Stripped of their cultural pride and entire existence, my people were forced to assimilate into a dominant culture. Their culture was no longer acceptable, the loss of ceremonies, their means of survival restricted, no longer allowed to hunt or gather. Their language was forbidden to be spoken, and like children at residential school, they were beaten when speaking Cree.

The trauma that my people and other Indigenous people experienced at the hand of a dominant oppressor has continued to be lived out into the generations; it is clearly observed in the pain of my people. Unresolved trauma and unresolved grief are the scares of the oppressors!

My mother told me stories of when she was a little girl and not being allowed to go into public places because she was Native. My mother spent most of her life in Residential school, from 5 years old to 18 years of age. These traumatic experiences stayed with my mother all her life, the abuse that she experienced throughout her years at residential school; I witnessed her pain many times throughout my life, and I would watch my mother’s body slouch over in an oppressed manner when treated with ridicule, as she was being racially demeaned as a Cree woman. I have many stories of witnessed treatment throughout my life that had an absolute direct effect on my own life and the lives of my family. The continued intergenerational issues created a mental, intellectual, emotional and spiritual pandemic for the Indigenous peoples that is lived out today.

The outcomes of the cultural genocide and the direct assault on the Indigenous identity have rooted an atrocious outcome for the lives of Indigenous peoples. These outcomes can be witnessed in the Child Welfare Systems, our children are being removed from their families and raised in non-native homes, our institutions are overpopulated by indigenous people, our men, women, and children are in continual danger of going missing or murdered. Poverty and low quality of life are all outcomes of these deep roots. 

## 7. The Historic, Current and Ongoing State of Living for Canadian Indigenous People: Kristy Bergeron

This population has generally retained a tone of disparity. As Indigenous people, we have grown accustomed to the underlying widespread concepts that define our existence that entail mostly negative and derogatory opinions that are backed by found facts and statistics. According to government-collected statistics, we generally have lower life expectancies, higher disease rates, lower education, and employment rates, we mostly live below the poverty line in comparison to the rest of the Canadian population, and we are over-represented in the child welfare systems and in the judicial system.

According to Statistics Canada, “In 2016, there were 1,673,780 Aboriginal people in Canada, making up 4.9% of the population. A history of colonization, including residential schools (the last of which closed in 1996), work camps and forced relocation, is recognized for having profoundly impacted Indigenous communities and families. Indigenous peoples often experience social and institutional marginalization, discrimination, and various forms of trauma and violence- including intergenerational and gender-based violence. As a result, many Indigenous people experience challenging social and economic circumstances. These factors play a significant role in the overrepresentation of Indigenous people in the criminal justice system and as victims of crime.” [30].

Since the arrival of Westerners to North America, the relationship between the Canadian State and First Nations people has not so much changed, the creation of reserves becoming modern-day ghettos, the Indian Agents as Canada’s first form of policing of Aboriginal people, and Residential Schools have morphed into what we now call child welfare. There has always existed a policy-making power structure of elite individuals, which have a mandate to maintain order and stratification in Canada. So that the status quo hierarchy remains, where the government, policy makers, and fortuned stakeholders can be found at the top of that hierarchy with policies to protect their interests and police to enforce those interests, and then, of course, you can find the first people of the land marginalized and without the same privilege and opportunities. “The seat of rule, with the dominion of rule. The mere act of providing a model, a paragon, a faultless image of civilized existence” [31] (p. 40). There exists within Canada a group of people that have the knowledge and the power to produce discourses or perceptions, where they are, so to speak, models that other groups should try to replicate and are judged and discriminated against for being unable to duplicate their standards, beliefs or worldviews. This systemic issue has eliminated the opportunity for examining the credibility of differing streams of knowledge, such as oral tradition, traditional medicinal knowledge, and traditional ecological knowledge and the importance of sovereignty to the resilience of oppressed people groups.

Throughout the past 200 years in Canada, there have been many attempts to eliminate First Nations people, including the development of residential schools. The intention of the formation of Indian Residential schools was to re-socialize the First Nations children so that they were no longer able to identify with their Aboriginal roots and culture so that with every coming generation, we would become less and less Indigenous. The approach to eliminating Indians was to have them integrate into a Western way of living. Remove them from their families, prohibit the practice of their culture, forbid the use of their language and enforce their own. This theory was unsuccessful once implemented, and then later eradicated, but we now know that it has led to many other issues that affect people and society as a whole. Taking children away from their primary attachments and out of their communities was so traumatic that it has devastated people for generations to come and created a major issue of that being the inability of a lot of adults to parent their children the way we once did traditionally.

The broken and oppressed population of First Nations people has always been referred to as the “Indian problem.” The idea that taking away our culture and Indigenousness would assimilate us into larger society would in turn dissolve our rights to tribal lands (Canada) and integrate into larger society. That has not yet happened, our survival spirits would not easily allow that and with Charters of Rights and Freedoms that have granted all Canadians the rights to worship and practice their culture, it is now no longer illegal to engage in our own traditional spiritual practices as it was once so recently. We are becoming more intentional about reconnecting with our culture because it is what feels right, it is embedded in our entire beings, and it is what we need to become whole again. The inability to access health and wellness from an Indigenous perspective has proven to have negative impacts in all areas of functioning for Indigenous people: physical health, mental health, spiritual well-being, as well as emotional ability. “Interconnectedness is an Aboriginal holistic value present in an Aboriginal worldview. Their wellness characteristics have been identified mainly as an interconnectedness that ties their identity to all life experiences” [32] (p. 15)). Wellness is encompassing all four parts of a person’s being, holistically, if one part of the being is out of balance, all other aspects will also suffer.

Throughout all those struggles to break our spirits and to eliminate our culture, First Nations people have managed to persevere and have maintained a sense of solidarity using whatever mechanisms that suffice at the time; just mere coping and survival has been such an objective. Being safe, housed, and having food is sometimes a luxury as it is said that you can compare our living conditions to that of an underdeveloped country. Men, women, and children go missing at alarming rates, we are imprisoned at alarming rates, our children are still taken at alarming rates. However, we are still here. We use laughter to talk about serious issues that we may not otherwise be able to cope with if we did not normalize them and laugh about it. For instance, Don Burnstick (Cree Comedian, from Alexander First Nation, Alberta) jokes, “You might be a Red Skin if…” Comedians have themes related to poverty, violence, jail, and promiscuity. We have found other creative outlets to express the emotions that are hard to consciously put into words, including avenues such as art, music, and dance. Another way that we have managed to get through many really hard times is through substance abuse; a way that we have found we are able to self-medicate and disassociate from the pain that we have endured. It is not an effective or productive way to help to ease what we have experienced for the past 300 years, but we have utilized whatever means necessary to survive.

There is something inexplicable about not being able to practice your culture and spirituality in a way that you were inherently designed to. It is intangible, unmeasurable, and almost metaphysical in the way we need healing. There is no existing scientific method to measure trauma, nevertheless inter-generational trauma, but we know it exists, and we need to realize that conventional and standard treatment models have not been successful as agents of healing. Treatment needs to be all-encompassing where we can utilize both modern and traditional methods of healing for all four parts of our being so that we can begin dealing with our current state and our historic traumas. 

## 8. Let Us Restore, Revitalize, and Return to Our Indigenous Ways: Geralyn Dorothy Wright

We cannot live as hunters and gathers any longer that time has gone, but we can embrace our historical ancestry with honor and pride, when healed.

To build capacity to care for families, our communities, and ourselves.

To reconcile.

Our culture and our identity can be healed, and we can walk the red road with our historical oppressor as an equal. With the honest observations of the issues at hand and with a strategic plan and resources to begin the journey, it can be attained.

Indigenous peoples of the land governed themselves with creation and had medicines to heal themselves; these solutions are still available to the people.

Healing lodges.

Ceremonies.

Shamanic energy healing.

Our traditional ways of life are still accessible and belong to all Indigenous people of the land. Our identities as Indigenous people must be restored and revitalized to embrace ourselves with the confidence of who we are. For way too many years, we have been told that we are not capable, nor do we have value; all capacity ripped from our existence. These practices are ancient healing medicines that heal the whole being, as indicated on a medicine wheel: mental, emotional, physical and spiritual (Geralyn Dorothy Wright).

## 9. To See through My Eyes—A Poem, Geralyn Dorothy Wright

Many Eyes

Many Hearts

Many, many tears

 

The many roads walked

The many mountains climbed

A peak to be reached

 

But who am I?

Just another Cree woman

A woman with a desire, a woman with a dream

 

The toils and turmoil’s all around

They are strong, they are real

Will they ever stop?

 

The journey challenged

The temptation to give in

To just give up and be another statistic

 

The resistors that attempt to hold me back

The resistors that call my name

They are the ghosts of my past, I see them

I hear them, will they ever let me go?

(Geralyn Dorothy Wright)
